# Phase I Trial of TTI-101, a First-in-Class Oral Inhibitor of STAT3, in Patients with Advanced Solid Tumors

**DOI:** 10.1158/1078-0432.CCR-24-2920

**Published:** 2025-01-10

**Authors:** Apostolia M. Tsimberidou, David J. Vining, Sukeshi P. Arora, Sofia de Achaval, Jeffrey Larson, John Kauh, Carrie Cartwright, Rony Avritscher, Imran Alibhai, David J. Tweardy, Ahmed O. Kaseb

**Affiliations:** 1 Department of Investigational Cancer Therapeutics, The University of Texas MD Anderson Cancer Center, Houston, Texas.; 2 Department of Diagnostic Imaging, The University of Texas MD Anderson Cancer Center, Houston, Texas.; 3 Mays Cancer Center, The University of Texas Health Science Center at San Antonio, San Antonio, Texas.; 4 Tvardi Therapeutics, Inc., Sugar Land, Texas.; 5 Department of Interventional Radiology, The University of Texas MD Anderson Cancer Center, Houston, Texas.; 6 Department of Infectious Diseases, Infection Control, and Employee Health, The University of Texas MD Anderson Cancer Center, Houston, Texas.; 7 Department of Molecular and Cellular Oncology, The University of Texas MD Anderson Cancer Center, Houston, Texas.; 8 Department of Gastrointestinal Medical Oncology, The University of Texas MD Anderson Cancer Center, Houston, Texas.

## Abstract

**Purpose:**

Signal transducer and activator of transcription 3 is a transcription factor that is essential for the survival and immune sequestration of cancer cells. We conducted a phase I study of TTI-101, a first-in-class, selective small-molecule inhibitor of signal transducer and activator of transcription 3, in patients with advanced metastatic cancer.

**Patients and Methods:**

Patients were treated with TTI-101 orally twice daily in 28-day cycles at four dose levels (DL): 3.2 (DL1), 6.4 (DL2), 12.8 (DL3), and 25.6 (DL4) mg/kg/day (“3+3” design). Three TTI-101 formulations were used in a stepwise manner (NCT03195699).

**Results:**

Sixty-four patients were treated (median age, 63 years; male sex, 52%; median number of prior therapies, 3). No dose-limiting toxicities or fatal treatment-related adverse events (TRAE) were observed. Diarrhea (mostly grade 1/2) was the only TRAE observed in ≥30% of subjects. Five patients experienced grade 3 TRAEs that resolved. TTI-101 showed linear pharmacokinetics from DL1 to DL3, with the pharmacokinetics plateauing at DL3. The recommended phase II dose is 12.8 mg/kg/day (DL3). Of the 41 patients who were evaluable for response, five (12%) had confirmed partial responses (cPR) and 17 (41%) had stable disease. Three (18%) of the 17 patients with hepatocellular carcinoma had a cPR (median time to treatment failure, 10.6 months). Two other cPRs were noted in one patient with ovarian cancer and one patient with gastric cancer.

**Conclusions:**

TTI-101 was well tolerated. cPRs were observed across tumor types. The antitumor activity of TTI-101 monotherapy in patients with advanced, metastatic solid tumors is promising. A phase II study of TTI-101 in hepatocellular carcinoma is currently underway.

**Translational Relevance**

Signal transducer and activator of transcription 3 (STAT3) is a transcription factor at the critical intersection of signaling pathways that regulates gene networks integral to cancer cell survival and immune sequestration. In this first-in-human phase I study, TTI-101, a first-in-class, orally bioavailable, selective small molecule that binds to STAT3 and prevents STAT3-mediated transcriptional activity, was well tolerated. Confirmed partial responses were noted in patients with hepatocellular carcinoma (HCC) and other solid tumors that had relapsed or were refractory to standard treatment. STAT3 is distinct from the currently established targets in HCC. By targeting both intrinsic tumorigenesis and extrinsic immune suppression, TTI-101 showed promising antitumor activity in patients with HCC refractory to immune checkpoint inhibitors and anti-angiogenic agents. Phosphotyrosine STAT3 levels were decreased after TTI-101 treatment in all paired biopsies in which phosphotyrosine STAT3 was elevated at baseline. A phase II study of TTI-101 is underway in patients with HCC.

## Introduction

Signal transducer and activator of transcription 3 (STAT3) is a member of the STAT family of seven closely related proteins responsible for the transmission of peptide hormone signals from the extracellular surface of cells to the nucleus (1). STAT3 is a master regulator of most key hallmarks and enablers of cancer (2–5), including cell proliferation (6), apoptosis resistance (7), metastasis (2), immune evasion (8), tumor angiogenesis (9), epithelial–mesenchymal transition (10), DNA damage response (11), and the Warburg effect (12). *STAT3* is also a key mediator of oncogene addiction (13) and supports the self-renewal of tumor-initiating cancer stem cells, contributing to cancer initiation, maintenance, and relapse (14–16) in several tumor types.

STAT3 activity is increased in approximately 50% of all patients with cancer (17) because of naturally occurring *STAT3* mutations, as has been demonstrated in human inflammatory hepatocellular adenomas and large granular lymphocytic leukemia (18, 19), or, more commonly, because of the activation of signaling molecules upstream of STAT3, including receptor tyrosine kinases (e.g., EGFR), tyrosine kinase–associated receptors (e.g., IL-6 cytokine receptors, which are associated with Janus kinase or G protein–coupled receptors; refs. 20–23), and Src kinases (e.g., Src, Lck, Hck, Lyn, Fyn, or Fgr; refs. 22, 23). Cancers with elevated STAT3 activity include renal cell carcinoma, colorectal cancer, ovarian carcinoma, gastric carcinoma, breast cancer, lung cancer, hepatocellular carcinoma (HCC), pancreatic adenocarcinoma, and head and neck squamous cell carcinoma (3). High-STAT3 activity is associated with poor prognosis in cancer; thus, STAT3 is an attractive target for drug development. TTI-101 is a first-in-class, orally delivered, small-molecule inhibitor of STAT3 that has been developed as a potential treatment for cancers (24, 25). It inhibits STAT3 by binding tightly within its Src homology 2 domain, which prevents the recruitment of STAT3 to signaling complexes that contain activated tyrosine kinases, thereby preventing STAT3 activation via phosphorylation on Y705. Binding of TTI-101 to STAT3 also prevents STAT3 homodimerization.

TTI-101 has been shown to have limited toxicity in laboratory animals in Good Laboratory Practice toxicology studies. Pharmacokinetic (PK) and toxicology studies in rats and monkeys have demonstrated that TTI-101 administration provides excellent plasma exposure following oral administration; no toxicities were detectable on gross, microscopic, or clinical laboratory evaluations when TTI-101 was administered for 13 weeks in monkeys and for 26 weeks in rats.

Based on the promising results for TTI-101 in preclinical studies, its highly attractive target, and its safety in nonclinical studies, we conducted a first-in-human, phase I, multicenter, dose-escalation and -expansion study of TTI-101 in patients with advanced metastatic solid tumors to determine its safety and efficacy.

## Patients and Methods

Patients were identified and recruited from The University of Texas MD Anderson Cancer Center and The University of Texas Health Science Center at San Antonio. All patients were enrolled from December 5, 2017, to August 15, 2022. Eligible patients were at least 18 years of age, with a histologically confirmed diagnosis of a solid tumor and advanced metastatic disease that had progressed after standard therapy. Briefly, patients were included if they had adequate bone marrow (hemoglobin ≥9.0 g/dL, neutrophil count ≥1.0 × 10^9^/L, and platelets ≥75 × 10^9^/L), renal (creatinine clearance >40 mL/minutes using the Cockroft–Gault formula), and liver [total bilirubin <1.5 × upper limit of normal [ULN] and aspartate aminotransferase (AST)/alanine aminotransferase (ALT) <3 × ULN] functions. For patients with liver involvement, an AST/ALT ratio <5 × ULN was required. Patients with childbearing potential needed a negative pregnancy test and had to agree to use effective contraception. Patients had an Eastern Cooperative Oncology Group performance status of 0 to 1 with measurable disease per RECIST v1.1. Eligible patients with HCC had a histologically or radiologically confirmed diagnosis of locally advanced, inoperable, or unresectable HCC and a Child–Pugh score of A.

Key exclusion criteria included previous anticancer therapy within 28 days of day 1 of the study drug treatment, known active metastases in the central nervous system, and chronic hepatitis B virus infection.

The study was approved by the Western Institutional Review Board. The study was conducted in accordance with the Belmont Report and the US Common Rule. All the patients signed an informed consent document stating that they were aware of the investigational nature of the study. This study was registered at www.clinicaltrials.gov (Identifier: NCT03195699) and followed STROBE reporting guidelines. The detailed inclusion and exclusion criteria are available in the Supplementary Material S1.

### Treatment

**Figure 1.**
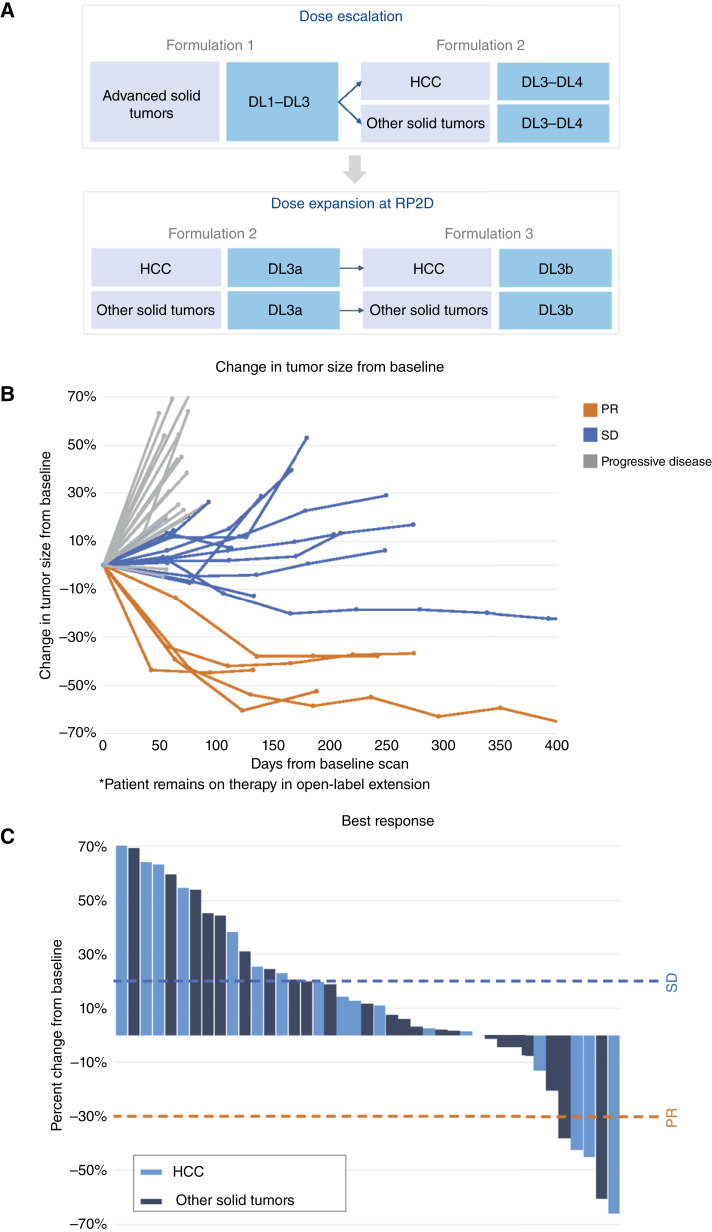
Study design and tumor response to TTI-101 monotherapy. **A,** Study design outlining the dose-escalation and dose-expansion phases. The dose-escalation phase involved advanced solid tumors across four DLs (DL1–DL4), with dose expansion at the RP2D for HCC and other solid tumors. Different TTI-101 formulations (F1, F2, and F3) were used to optimize delivery and tolerability. **B,** Spider plots depicting individual tumor responses to TTI-101 treatment over time. Each line represents a patient’s sum of target tumor response, with changes in tumor size from baseline measured at various time points. The plot illustrates the variability in response across patients and DLs. **C,** Waterfall plot showing the best response by RECIST criteria for patients treated with TTI-101 monotherapy. Bars represent the maximum reduction in the sum of target tumor size for each patient, categorized as a PR, SD, or progressive disease. Tumor types are indicated, highlighting the distribution of responses across HCC and other solid tumors.

Patients were treated with TTI-101 orally administered twice daily for 28-day cycles. The starting dose was determined using the US FDA Small Molecule Guidelines for the determination of the maximum recommended starting dose, which considers the no observed adverse effect level, human equivalent dose, and 10-fold safety factor. Patients were treated at the following escalating dose levels (DL): 3.2 (DL1), 6.4 (DL2), 12.8 (DL3), and 25.6 (DL4) mg/kg/day (Fig. 1A). The safety, tolerability, and PK of each DL were reviewed by the safety review committee, which was composed of the principal investigators, medical monitor designated by the sponsor, and PK expert. A unanimous decision was required for dose escalation. An independent Data Safety Monitoring Board reviewed patient safety data and dose-escalation decisions and identified no serious safety issues or conflicts of interest in the conduct of this study.

Toxicity was assessed in accordance with the Cancer Therapy Evaluation Program active version of the NCI Common Terminology Criteria for Adverse Events v5.0. Treatment was continued until disease progression, unacceptable toxicity, or study withdrawal.

Three formulations of TTI-101 were used in this study, beginning with formulation 1 (F1), which used Labrasol, a nonionic surfactant-solubilizing excipient, and encapsulation. During the study, the capsule burden became a limiting factor for drug administration at higher dosages; as a result, F2 was implemented, which used a self-emulsifying drug delivery system. F3, a spray-dried dispersion tablet, was developed to further reduce the number of capsules or pills.

### Patient monitoring

Patients were monitored by physical examination (including vital signs and performance status) before the initiation of therapy, weekly, and on day 1 of each cycle. Safety and tolerability parameters included adverse events (AE), treatment-related AEs (TRAE), laboratory parameters, Eastern Cooperative Oncology Group performance status, vital signs, echocardiogram, electrocardiogram, physical examination, and recording of concomitant medications. PK parameters were estimated from blood plasma concentrations of TTI-101 before cycle 1, day 1 morning dosing and serially up to 12 hours after dosing. The response was assessed approximately every 6 weeks after the start of treatment.

### Primary endpoints

The primary endpoints of the study were the maximum tolerated dose (MTD) or recommended phase II dose (RP2D), dose-limiting toxicities (DLT) and tolerability of TTI-101, and the PK of TTI-101 in plasma following oral administration.

#### Determination of MTD

MTD was defined as the highest dose at which ≤1/6 of the patients experienced DLTs in the first treatment cycle. If an MTD was not reached, the RP2D was determined by evaluating safety and PK endpoints at a DL below the maximum delivered dose.

#### DLTs and tolerability of TTI-101

DLTs were defined as any of the following within the first cycle (28 days) of treatment with TTI-101: any death not clearly due to an underlying disease or extraneous cause; any grade 3 or 4 nonhematologic toxicity, as defined in the most current version of the Common Terminology Criteria for Adverse Events, even if expected and believed to be unrelated to the study medications (except nausea and vomiting, electrolyte imbalances that are responsive to appropriate regimens, or alopecia); any grade 3 nausea, vomiting, or diarrhea that lasted >72 hours despite maximum supportive care; any grade ≥3 electrolyte abnormality that lasted >72 hours, was clinically complicated, or did not respond to conventional medical interventions; hepatocellular injury (elevated ALT or AST ≥3-fold the upper limit of normal) clearly attributed to the study drug (the Hy law); neutropenic fever; grade 3 thrombocytopenia with significant bleeding; or grade 4 thrombocytopenia for more than 7 days. TEAEs were coded using the Medical Dictionary for Regulatory Activities (v23.1.).

#### PK of TTI-101

K_2_EDTA human plasma test samples were delivered under frozen conditions on dry ice to the bioanalytic laboratory (Frontage Laboratories, formerly Biopharmaceutical Research Institute, Inc.) to determine the TTI-101 concentration. The results were validated using an LC/MS-MS assay. The validated lower limit of quantification for TTI-101 was 5.00 ng/mL, and the upper limit of quantification was 1,000 ng/mL. Chromatographic integration was performed using MassLynx v4.0 (Waters Corporation; RRID: SCR_014271). Regression and statistical analyses were performed using the Thermo Scientific Watson Bioanalytic LIMS System (v7.4.0.0; RRID: SCR_002974) and Microsoft Excel 2007 or higher. The PK parameters of TTI-101 were determined following the first dose in F1- and F2-treated patients. PK evaluation was performed using Phoenix WinNonlin (v8.3; RRID: SCR_024504).

PK parameters, including the maximum observed plasma concentration, trough plasma concentration, half-life, and area under the plasma concentration–time curve (AUC), were estimated using standard noncompartmental methods.

### Secondary endpoints

The secondary endpoints included the pharmacodynamics of the TTI-101, both before and during treatment, and clinical outcomes.

#### Pharmacodynamics of phosphotyrosine STAT3

Anti–phosphotyrosine (pY) STAT3 IHC staining was performed on 4-μm, unstained, formalin-fixed, paraffin-embedded tissue sections using a Leica Bond III autostainer (Leica Biosystems; RRID: SCR_023957). For anti–pY-STAT3 immunofluorescence, a Leica Bond RX autostainer (Leica Biosystems; RRID: SCR_023957) was used. Following deparaffinization and rehydration, heat-induced epitope retrieval was performed using Tris-EDTA. A primary anti–human pY-STAT3 (Tyr705) antibody (clone D3A7, Cell Signaling Technology; RRID: SCR_002071) was used. For IHC, primary antibody detection was performed using a commercial polymer system (Bond Polymer Refine Detection, Leica Biosystems; RRID: SCR_023603), and stain development was achieved. Counterstaining was performed with hematoxylin. Tumor sections were stained with anti–phopho-STAT3 IHC and scanned with an Aperio A2s scanner (RRID: SCR_021256) to perform computerized automated image analysis of pY-STAT3. The images were stored and organized using Aperio eSlide Manager, v12.4.3.5008, and Aperio ImageScope (v12.1.0.5029; RRID: SCR_020993) was used for viewing and analysis. The algorithms used were tuned versions of the Aperio’s nuclear algorithm, v9.2. For immunofluorescence, the fluorescence reagents DAPI (Abcam; RRID: SCR_012931) and Opal 520 (Akoya; RRID: SCR_023774) were used. All immunofluorescence-stained sections were scanned with the NanoZoomer-HT v2.0 image system (Hamamatsu Photonics; RRID: SCR_017105) at 40× magnification. Representative images were analyzed using the HALO platform (Indica Labs; RRID: SCR_018350). Necrotic and stromal areas were excluded from the study.

Image analysis was performed to calculate the percentage of positive tumor cell nuclei in the hotspot, along with the average percentage of positive tumor cell nuclei in the entire tumor area, both of which were reported by a licensed pathologist in the pathology report.

To capture the staining intensity and the proportion of tumor cells stained for both IHC and immunofluorescence analyses, we calculated the *H*-score using the following formula: (1 × percentage of tumor cells with staining intensity 1) + (2 × percentage of tumor cells with staining intensity 2) + (3 × percentage of tumor cells with staining intensity 3). The resulting *H*-scores ranged from 0 to 300, as previously reported (26).

The pharmacodynamics of TTI-101 in patient tumors were assessed by measuring pY-STAT3 levels at baseline and after treatment. Paired *H*-scores were compared before and during the TTI-101 treatment.

#### Clinical outcomes

The clinical outcomes evaluated for the efficacy population included the best overall response (BOR), as determined by the percentages of participants with the following responses: complete response (CR), partial response (PR), stable disease (SD), progressive disease, and inability to assess, as per RECIST v1.1. The overall response rate (ORR) was calculated as the proportion of participants with confirmed CR or confirmed PR (cPR) as the BOR per RECIST v1.1. The clinical benefit rate was calculated as the proportion of participants with confirmed CR, cPR, or SD as the BOR per RECIST v1.1. Time to treatment failure (TTF) was measured from the date of treatment initiation to failure due to disease progression, AE, or death, whichever occurred first.

### Exploratory endpoints

Exploratory outcomes included the association between biomarkers and antitumor efficacy and survival outcomes, clinical outcomes related to liver fibrosis in patients with HCC, and the effects of food on TTI-101 bioavailability in patient subgroups.

#### Biomarkers and tumor molecular profiling

In patients with HCC, an exploratory retrospective analysis was performed to compare molecular markers among evaluable patients with a tumor molecular profile available from their medical history. Patients with available data were grouped according to the antitumor efficacy of TTI-101 and common mutations.

#### Viral versus nonviral etiology in patients with HCC

In patients with HCC, an exploratory analysis was performed to compare viral etiology (history of viral hepatitis B or C) with nonviral etiology (no history of viral hepatitis).

#### Effects of food on bioavailability

Descriptive statistics were used to characterize the effect of food on the bioavailability of TTI-101, including PK and safety parameters, among patients receiving F1-F3. To compare the fed and fasting systemic exposures, all available data were used to generate mean plasma concentrations of TTI-101 at each time point, including in the absence of complete time-point curves under both fed and fasting conditions. To compare the exposure to TTI-101 between fed and fasting patients and to compare the PK parameters between F2 and F3 patients, the plasma concentrations of the fasting dataset were normalized by subtracting the time zero (predose) mean concentration from each subsequent postdose concentration.

### Statistical considerations

In the dose-escalation part of the study, a “3+3” design was used without allowance for intrapatient dose escalation. The expansion phase included patients with advanced solid malignancies treated with TTI-101 at the RP2D determined in the dose-escalation phase of the study. Safety was summarized using descriptive statistics for all patients who received at least one dosage of the study drug. The PK results were estimated using standard noncompartmental methods.

Response was assessed in patients who completed at least two cycles of treatment (42 days after the initiation of treatment; efficacy-evaluable population). Descriptive statistics were used to summarize exploratory endpoints. All statistical analyses were performed using SAS, version 9.4 or higher (RRID: SCR_008567).

### Data availability

The availability of data from this clinical trial is subject to applicable local and federal regulations and ethical guidelines. Data will be available upon reasonable request to the corresponding author. Efforts will be made to facilitate timely and transparent responses to qualified inquiries.

## Results

### Patients and treatment

Sixty-four patients who enrolled in the study from December 2017 to September 2022 were treated with TTI-101 at two US sites. The patient demographics and key baseline disease characteristics are presented in Table 1 and Supplementary Table S1. The most common cancer type was HCC (*n* = 22), followed by breast and colorectal cancers (*n* = 7 each). The median number of prior systemic therapies was three (range, 1–9). As of the date of the last follow-up (May 1, 2024), 63 patients had discontinued treatment for the following reasons: 41 (65%) had disease progression, 10 (16%) had AEs, 10 (16%) withdrew consent [owing to poor tolerance of the study drug (*n* = 6), AEs unrelated to the study drug (*n* = 2), preference for treatment elsewhere (*n* = 1), or decline in performance status (*n* = 1)], and two (3%) patients had poor adherence to the protocol procedures. One patient transitioned from an active study to an open-label extension and continued therapy.

**Table 1.** Demographic and clinical characteristics at baseline (*n* = 64).

Characteristic	Result
Age, years, mean (SD)	61 (12)
Sex, *n* (%)	
Female	31 (48)
Male	33 (52)
ECOG performance status, *n* (%)	
0	7 (11)
1	57 (89)
Cancer type, *n* (%)	
HCC	22 (34)
Breast cancer	7 (11)
Colorectal cancer	7 (11)
Neuroendocrine carcinoma	3 (5)
Melanoma	2 (3)
Non–small cell lung cancer	2 (3)
Ovarian cancer	2 (3)
Pancreatic cancer	2 (3)
Other (one patient each)^a^	17 (27)
Initial stage at diagnosis, *n* (%)^b^	
0	1 (2)
1	2 (3)
2	4 (6)
3	24 (38)
4	19 (30)
Current stage, *n* (%)	
3	7 (11)
4	53 (83)
Median no. of prior systemic therapies (range)	3.5 (1–9)

Abbreviations: ECOG, Eastern Cooperative Oncology Group.

a Other includes mixed histology hepatocellular/cholangiocarcinoma, cholangiocarcinoma, neuroendocrine carcinoma (ovary), melanoma (uveal), lung cancer, cervical cancer, endometrial cancer, esophageal cancer, gastric cancer, parotid cancer, sarcoma (NOS), leiomyosarcoma, synovial sarcoma, thymoma, papillary thyroid cancer, tonsil cancer, and vulva cancer.

b Fourteen patients had missing initial stage at diagnosis.

### Safety and tolerability

Overall, 63 (98%) of the 64 patients experienced an AE. The most common AEs were diarrhea [*n* = 33 (52%)], fatigue [*n* = 26 (41%)], and nausea [*n* = 20 (31%)]. Table 2 shows the TRAEs that were observed in >10% of patients and may have been related to TTI-101. Overall, 70% of the patients experienced any grade of TRAE, whereas 25% experienced grade ≥3. No DLTs or fatal TRAEs were observed. Diarrhea, mostly grade 1/2, was the only TRAE observed in ≥30% of the patients. One patient experienced transient grade 4 hyperglycemia that was resolved with insulin and metformin. Five patients experienced grade 3 TRAEs that resolved. Ten patients (15%) experienced a grade ≥3 TRAE that resulted in drug interruption or withdrawal. A higher proportion of patients treated with DL4 experienced TRAEs and at higher grades. F3 (*n* = 7) was better tolerated than F1 (*n* = 15) and F2 (*n* = 47).

**Table 2.** Summary of TRAEs reported in >10% of patients treated with TTI-101 monotherapy.

TRAE, any grade	All DLs		DL1 (3.2 mg/kg/day)		DL2 (6.4 mg/kg/day)		DL3 (12.8 mg/kg/day)		DL4 (25.6 mg/kg/day)	
	*N* = 64		*N* = 4		*N* = 3		*N* = 47		*N* = 10	
	≥3	All	≥3	All	≥3	All	≥3	All	≥3	All
Any TRAE	16 (25)	45 (70)	0	1 (25)	0	3 (100)	13 (28)	35 (74)	3 (30)	6 (60)
Diarrhea	9 (14)	30 (47)	0	0	0	2 (67)	7 (15)	22 (47)	2 (20)	6 (60)
Nausea	2 (3)	12 (19)	0	0	0	1 (33)	2 (4)	11 (17)	0	0
Fatigue	0 (0)	10 (6)	0	0	0	1 (33)	0	8 (17)	0	1 (10)
ALT increased	4 (6)	8 (13)	0	1 (25)	0	0	3 (6)	6 (13)	1 (10)	1 (10)
AST increased	4 (6)	7 (11)	0	0	0	0	4 (9)	6 (13)	0	1 (10)

Data are presented as the number of patients and percentage (%). TRAEs were related to the drug and included “definitely related,” “possibly related,” and “probably related” AEs.

### PK of TTI-101

The PK results are presented in Table 3. TTI-101 showed linear PK from DL1 to DL3, whereas the increase in systemic exposure from DL3 to DL4 was less than proportional with the increase in dosage. The minimum concentration of DL3 was above the expected STAT3 IC_50_ for cell growth inhibition (25). A significant increase in exposure was observed (77% increase by AUC/dose × body weight in kgs) when transitioning from the F1 formulation to the F2 formulation at RP2D. No differences were noted in exposure to TTI-101 between the fed and fasting subgroups receiving the F2 dose formulation on cycle 1, day 1 and cycle 2, day 1, respectively. Intrapatient crossover data for the F2 and F3 formulations on consecutive days demonstrated that F3 patients had the same exposure to TTI-101 as patients receiving the F2 formulation.

**Table 3.** PK parameters of TTI-101 in plasma.

Formulation	DL	Dose (mg/kg)	*C*_max_ (ng/mL)	*C*_max_/dose (ng/mL/mg)	AUC_0–last_ (ng × hours/mL)	AUC_0–last_/dose (ng × hours/mL/mg)	*t*_1/2_ (hours)
F1	DL1 (3.2 mg/kg/day)	1.74 (0.07)	1,090 (247)	620 (137)	6,360 (976)	3,640 (448)	8.55 (3.40)
	*N* = 4						
	DL2 (6.4 mg/kg/day)	3.25 (0.09)	2,460 (707)	755 (198)	14,800 (4,380)	4,530 (1,250)	5.61 (2.49)
	*N* = 3						
	DL3 (12.8 mg/kg/day)	6.52 (0.17)	2,580 (575)	395 (81)	19,100 (6,400)	2,920 (954)	11.9 (8.33)
	*N* = 8						
F2	DL3a (12.8 mg/kg/day)	6.73 (0.44)	6,510 (4,200)	972 (621)	34,900 (21,700)	5,190 (3,160)	5.91 (4.18)
	*N* = 10						
	DL4 (25.6 mg/kg/day)	13.3 (1.02)	9,940 (5,790)	752 (431)	50,700 (15,800)	3,850 (1,220)	8.28 (6.71)
	*N* = 10						
F2 food effect	F2 fed (12.8 mg/kg/day)	6.53 (0.32)	4,660 (2,270)	719 (359)	21,200 (10,700)	3,250 (1,600)	NC
	*N* = 25						
	F2 fasted (12.8 mg/kg/day)	6.51 (0.29)	5,940 (2080)	913 (315)	22,300 (9,010)	3,430 (1,350)	NC
	*N* = 24						
F2 and F3 crossover	F2 (400 mg/day)	5.63 (1.10)	7,340 (8,570)	1,180 (1,290)	24,000 (26,400)	3,870 (3,920)	NC
	*N* = 2						
	F3 (400 mg/day)	5.63 (1.10)	4,230 (4,350)	688 (638)	21,900 (15,800)	3,590 (3,110)	NC
	*N* = 2						

Data are presented as mean (SD) unless otherwise indicated.

Abbreviations: AUC_0–last_, AUC from time zero to last time point, in which the last time point was 12 hours for F1 and F2 and 8 hours for the F2 food effect and the F2 and F3 crossover evaluations; *C*_max_, maximum concentration; *t*_1/2_, elimination half-life; NC, not calculated.

### Determination of the RP2D

As no DLTs were observed during DL1-DL4, a review of the cumulative safety and PK data for DL1-DL4 was conducted to determine the RP2D. A higher proportion of patients experienced TRAEs and at a higher grade at DL4 compared with that at DL3. In addition, exposures were dose-proportional from DL1 through DL3; however, dose-normalized exposures were lower at DL4 than at DL3. No differences in safety or PK were noted between patients with HCC and those with other solid tumors. The RP2D was therefore determined to be at DL3 (12.8 mg/kg/day) for both the HCC and other solid tumor cohorts.

### Pharmacodynamics of pY-STAT3

Pre- and on-treatment paired tumor biopsies evaluable for pharmacodynamic analysis were available for 10 patients. Eight patients had elevated baseline pY-STAT3 *H*-scores, defined as >30 (of a total score of 300). All eight patients demonstrated a decrease in the *H*-score at the follow-up biopsy (approximately 6 weeks after initiating treatment), with a median decrease of 55%. Among the three patients who demonstrated a clinical benefit, the median decrease in the *H*-score was 79%.

### Clinical outcomes

**Figure 2.**
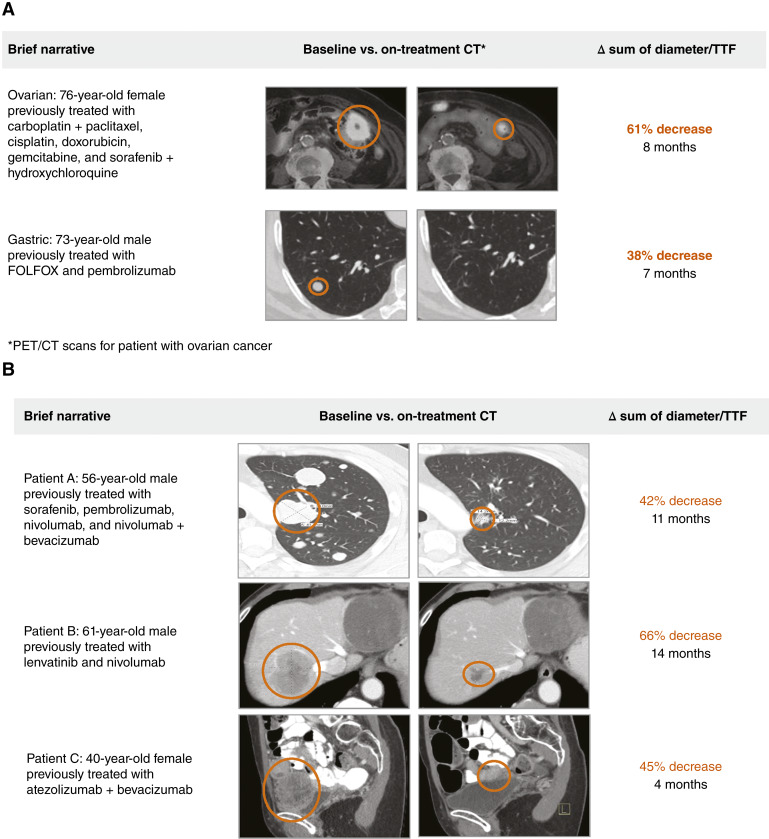
cPRs to TTI-101 monotherapy in patients with ovarian and gastric cancers. **A,** The patient with ovarian cancer (76-year-old female) achieved a 61% decrease in the sum of target tumor diameter with a TTF of 8 months. The patient with gastric cancer (73-year-old male) experienced a 38% reduction in the sum of target tumor size with a TTF of 7 months. PET/CT scans demonstrate sample target tumor regression from baseline to on-treatment. **B,** cPRs in patients with HCC treated with TTI-101 monotherapy. Patient A (56-year-old male) achieved a 42% reduction in the sum of target tumor size as BOR with an 11-month TTF, patient B (61-year-old male) experienced a 66% reduction with a TTF of 14 months, and patient C (40-year-old female) showed a 45% reduction with a TTF of 4 months. These responses illustrate the potential efficacy of TTI-101 in heavily pretreated patients with HCC.

Of the 41 patients who were evaluable for response, five (12%) had cPRs as the BOR and 16 (41%) had SD (Fig. 1B; Table 4). Among the 17 patients with HCC, three achieved cPR (ORR: 18%) with a median TTF of 10.6 months (Fig. 1C), and six (35%) had SD. The other two cPRs occurred in one patient with ovarian cancer and in one patient with a gastric tumor. Of the two evaluable patients with ovarian cancer, one had a cPR (best response, 61% reduction in the sum of target lesions; TTF, 7.1 months) and the other had SD (TTF duration, 3.9 months). The only evaluable patient with a gastric tumor had cPR (best response, 38% reduction in the sum of target lesions; TTF, 8 months; Fig. 2A).

**Table 4.** Best response to TTI-101 treatment per RECIST v1.1.

BOR	DL										
	1		2		3		4				
	(3.2 mg/kg/day) (*N* = 3)		(6.4 mg/kg/day) (*N* = 3)		(12.8 mg/kg/day) (*N* = 30)		(25.6 mg/kg/day) (*N* = 5)		All DLs		
	HCC	Non-HCC	HCC	Non-HCC	HCC	Non-HCC	HCC	Non-HCC	Total HCC	Total non-HCC	Total
	(*N* = 0)	(*N* = 3)	(*N* = 2)	(*N* = 1)	(*N* = 13)	(*N* = 17)	(*N* = 2)	(*N* = 3)	(*N* = 17)	(*N* = 24)	(*N* = 41)
PR	0	0	1 (50)	0	2 (15)	2 (12)	0	0	3 (18)	2 (8)	5 (12)
SD	0	2 (67)	0	0	6 (46)	9 (53)	0	0	6 (35)	11 (46)	17 (41)
Progressive disease	0	1 (33)	1 (50)	1 (100)	5 (38)	6 (35)	2 (100)	3 (100)	8 (47)	11 (46)	19 (46)

The efficacy population and secondary analysis set included patients with a follow-up on-study tumor assessment of at least 42 days following cycle 1, day 1.

Seventeen (41%) of the 41 evaluable patients had SD with a median TTF of 3.9 months (range, 1.4–11.3 months). At the last follow-up (May 2024), one patient with melanoma remained on treatment with TTI-101, with SD for >20 months (22% decrease in the overall sum of tumors).

The overall clinical benefit rate was 54% (HCC, 53%; non-HCC, 54%). All patients with HCC who experienced cPR had disease that was refractory to immunotherapy and antiangiogenic agents; overall, the median number of prior systemic therapies was two. All cPRs in patients with HCC were observed during the first restaging scan (Fig. 2B).

### cPRs in patients with HCC

**Patient A** (a 56-year-old man) had an HCC tumor that previously failed to respond to treatment with sorafenib, pembrolizumab, nivolumab, and nivolumab combined with bevacizumab. After treatment with TTI-101 monotherapy (DL2), the best response was a 42% reduction in the sum of the target lesions, with a TTF of 10.6 months.

**Patient B** (a 61-year-old man) had progressive disease after treatment with lenvatinib and subsequently with nivolumab before initiating treatment with TTI-101 at DL3. His best response was a 66% reduction in the sum of the target lesions, sustained with a TTF of 13.9 months; however, the patient discontinued therapy because of the progression of a nontarget lesion. The patient was subsequently treated with a combination of atezolizumab and bevacizumab within 30 days of discontinuation of TTI-101 and demonstrated a new response after 2 months, with a decrease in target and nontarget lesions.

**Patient C** (a 40-year-old woman) had a disease that was refractory to atezolizumab and bevacizumab before initiating TTI-101 at DL3. Her best response was a 45% reduction in the sum of the target lesions, with a TTF of 4.2 months, despite several dose interruptions due to TRAEs (gastrointestinal disorders) or TEAEs unrelated to treatment (COVID-19).

### Response by etiology in patients with HCC

Of the 17 evaluable patients with HCC, 10 (59%) had viral etiology and seven (41%) had nonviral etiology. Notably, three of the seven patients with nonviral etiology had cPRs and one had SD (ORR: 43%; disease control rate: 57%). Of the 10 patients with viral etiology, five had SD (clinical benefit rate, 50%), and no objective responses were noted.

### Tumor molecular profiling

Retrospective tumor molecular profiling data were available for 44 patients. Because of the variability in tumor type and advanced metastatic disease status, numerous mutations have been observed. A trend was noted between p53 mutations and TTI-101 response in patients with HCC. Among 10 patients with HCC who were evaluable for response and had known p53 mutational status, 86% (six of seven) of those with mutant p53 experienced a clinical benefit (two PRs and four SDs) compared with none of the patients with wild-type p53 tumors.

## Discussion

In this first-in-human phase I study, TTI-101 was well tolerated and demonstrated clinically meaningful antitumor activity in patients with advanced, metastatic solid tumors that had progressed after standard therapy. No DLTs were noted at any of the four DLs tested. Three formulations of TTI-101 were used. F3 was better tolerated than F1 and F2. TTI-101 showed linear PK for DL1-DL3, with a less-than-dose-proportional increase at DL4. The RP2D was determined to be 12.8 mg/kg/day (DL3) for the HCC cohort and the other solid tumor cohort.

Given the role of STAT3 as a central mediator of human diseases, a pharmacologic strategy that disrupts STAT3 activity in patients has been a long-sought-after goal. STAT3 is a transcription factor that is phosphorylated at a single tyrosine residue (Y705), resulting in its homodimerization and translocation to the nucleus, where it initiates a transcriptional program that is critical for inflammation, fibrosis, and oncogenesis. Notably, STAT3 also plays a key role in mitochondrial oxidative phosphorylation mediated by serine 727 phosphorylation. Serine 727–phosphorylated STAT3 interacts with mitochondrial partner proteins to promote mitochondrial ATP synthesis. Importantly, because of its precise binding to the Src homology 2 domain of STAT3, TTI-101 does not interfere with serine 727 phosphorylation or block the mitochondrial function of STAT3, unlike other STAT3 inhibitors (27). Consequently, TTI-101 did not have an impact on mitochondrial function in preclinical toxicology studies in a healthy volunteer population of 41 patients or in this trial, in which no patient experienced peripheral neuropathy or lactic acidosis.

Of the 41 patients who were evaluable for response, five (12%) had PRs and 16 (41%) had SD. Within the cohort of 24 patients with other solid tumors, two evaluable patients with ovarian cancer demonstrated reductions in tumor (one patient had a cPR and the other had SD), and the only patient enrolled with a gastric tumor had a cPR. Both patients with cPR were heavily pretreated.

In the patients with HCC, the ORR was 18% and the clinical benefit rate was 53%, with a median TTF of 10.6 months. This level of antitumor activity is encouraging in a patient population with refractory HCC. Although there are limited data with regard to the expected response rates following disease progression on two or more lines of systemic therapy, an ORR of <5% may be expected because many of the currently approved agents have overlapping mechanisms of action, such as antiangiogenics (bevacizumab and ramucirumab), immune checkpoint inhibitors (atezolizumab, durvalumab, nivolumab, and pembrolizumab), and multitargeted kinase inhibitors (sorafenib, lenvatinib, regorafenib, and cabozantinib; refs. 28–31).

Notably, in patient B, the response to combined atezolizumab and bevacizumab within 30 days of discontinuation of TTI-101 suggests a role for TTI-101 in re-sensitizing the tumor to immune checkpoint inhibitor therapy. STAT3 is unique and distinct from the currently established targets in HCC. By targeting both intrinsic tumorigenesis and extrinsic immune suppression using TTI-101, this study demonstrated the clinical activity in patients with HCC that was refractory to immune checkpoint inhibitors and antiangiogenic agents.

Our trial has limitations common to many oncology phase I trials, including enrollment of patients with a variety of heavily pretreated advanced malignancies and a complexity of comorbidities and molecular alterations that may affect the tolerability and antitumor activity of TTI-101. Trial limitations specific to our trial include limited PK data and limited safety information in patients with HCC with liver dysfunction. Also, TTI-101 exposure data are currently limited to the time period following initial TTI-101 administration, but this will be addressed by the collection of PK samples at a steady state in the ongoing phase II trials. With regard to safety data in patients with liver dysfunction, our data should be interpreted cautiously, as enrollment was limited to patients with a Child–Pugh score of A. This gap will be addressed in an ongoing HCC trial which allows the enrollment of patients with Child–Pugh scores of A as well as B7; however, the administration of TTI-101 to patients with more severe liver dysfunction will need to be addressed in future trials specifically evaluating organ dysfunction.

As part of an exploratory analysis, the etiology of HCC was investigated; all three patients with HCC who achieved cPR had nonviral etiologies. Reports have shown differentiated gene expression profiles for various HCC etiologies; molecular classification of HCC has helped to identify a subgroup of HCC tumors with a highly activated IL6–JAK–STAT signaling pathway that are more commonly associated with nonalcoholic steatohepatitis and alcoholic steatohepatitis (32). A reliance on the IL6–JAK–STAT pathway in the subgroup of nonviral-associated HCC could potentially explain our clinical findings. The mechanism by which TTI-101 treatment leads to response in these patients is being explored further.

The levels of pY-STAT3 were decreased in all paired biopsies in which pY-STAT3 was elevated at baseline. The largest decline in the pY-STAT3 *H*-score (mean decrease, 79%) was observed in the three patients who experienced a clinical benefit. In addition to study-specific tumor biopsies, a retrospective analysis of tumor mutation status revealed a trend toward clinical benefits in patients with HCC harboring mutated *p53*. Of the 10 patients with tumors with available *p53* mutation status, seven were reported to have mutant* p53*, including six patients who had clinical benefit with TTI-101 monotherapy. Conversely, all three patients with wild-type p53 showed progressive disease. Other investigators have demonstrated that mutant *p53* promotes tumorigenesis, metastasis, and invasion through sustained activation of *STAT3*. Furthermore, ovarian, breast, and prostate cancer cell lines which harbor *p53* mutations are also constitutively active for *STAT3* (33). Our hypothesis is that TTI-101 was particularly effective in the mutant *p53* population because *STAT3* activation was a key oncogenic driver and these tumors may have been preferentially sensitive to STAT3 inhibition. The correlation between clinical benefits and *p53* mutation status will be further investigated in ongoing clinical trials using TTI-101.

In conclusion, TTI-101 was well tolerated, with no DLTs noted; cPRs were observed across tumor types. The antitumor activity of TTI-101 monotherapy in advanced metastatic solid tumors is promising. A phase II clinical trial of TTI-101 is underway that is examining TTI-101 in patients with locally advanced or metastatic, unresectable HCC (NCT05440708) in three arms: TTI-101 in combination with atezolizumab plus bevacizumab as first-line therapy, TTI-101 in combination with pembrolizumab as second-line therapy, and TTI-101 as monotherapy as third-line therapy.

## Supplementary Material

Supplementary File S1 Supplementary File S1. Patient Eligibility.

Supplementary Table S1 Supplementary Table S1. Representativeness of Study Participants.
