# Supplementary material for: Phase I Trial of TTI-101, a First-in-Class Oral Inhibitor of STAT3, in Patients with Advanced Solid Tumors
Source: Clin Cancer Res. 2025 Jan 10;31(6):965–74. doi: 10.1158/1078-0432.CCR-24-2920 (PMC11911802; doi:10.1158/1078-0432.CCR-24-2920)
Supplement: Supplementary File S1 — Patient Eligibility. [file ccr-24-2920_supplementary_file_s1_suppsf1.docx]

# PATIENT ELIGIBILITY

## Inclusion Criteria

All of the following inclusion criteria must be fulfilled for eligibility:

1. Age ≥18 years;
2. For patients with solid tumors (not unresectable HCC): Patients with histologically confirmed diagnosis of locally advanced, inoperable, metastatic and/or treatment refractory solid tumors for whom there are no available therapies that will confer clinical benefit;
3. For patients with unresectable HCC: Patients with histologically or radiographically (per Liver Imaging Reporting and Data System - LI-RADS) confirmed diagnosis of locally-advanced, inoperable, unresectable HCC who have failed first and second lines of therapy and Child-Pugh is A or beyond second line if performance status is preserved and Child-Pugh is A.
4. Eastern Cooperative Oncology Group Performance (ECOG) status 0-1;
5. Hemoglobin ≥9.0 g/dL, neutrophil count ≥1.0 x 10^9^/L, platelets ≥75 x 10^9^/L;
6. Adequate renal function capability, as calculated by creatinine clearance >40 mL/min using the Cockroft-Gault formula.
7. Adequate liver function defined as total bilirubin <1.5 x ULN, and aspartate aminotransferase (AST)/alanine aminotransferase (ALT) <3 x ULN. For subjects with liver involvement, AST/ALT <5 x ULN.
8. Measurable disease using clinically appropriate criteria for the type of malignancy, RECIST v1.1 for solid tumors;
9. Negative pregnancy test at the screening visit for women of childbearing potential, defined as female subjects after puberty unless they have been postmenopausal for at least two years, are surgically sterile, or are sexually inactive and will remain so for the course of the trial;
10. Willingness to avoid pregnancy and breast feeding beginning two weeks before the first TTI-101 dose and ending three months after the last trial treatment. Male subjects with female partners of childbearing potential and female subjects of childbearing potential must use adequate contraception in the judgment of the Investigator, such as a two—barrier method or a one—barrier method with spermicide or intrauterine device during trial treatment dosing and for 3 months after the last dose of the study. Birth control pills are not considered an acceptable form of birth control for this study.
11. Ability to read and understand the informed consent form and willingness and ability to give informed consent and demonstrate comprehension of the trial before undergoing any trial activities.

## Exclusion Criteria

Subjects are ineligible to enroll in this trial if they fulfill any of the following exclusion criteria:

1. Previous therapy with:
   1. Standard therapy including chemotherapy, immunotherapy, biologic therapy, or any other anticancer therapy within 28 days (or five elimination half-lives for non-cytotoxic drugs, whichever is shorter) of Day 1 of trial drug treatment (6 weeks for nitrosoureas or mitomycin);
   2. Any investigational agent within 28 days of Day 1 of trial drug treatment or 5 half-lives for a small molecule/targeted therapy;
2. Extensive prior radiotherapy on more than 30% of bone marrow reserves, or prior bone marrow / stem cell transplantation within 5 years from enrollment; Ongoing toxicity (except alopecia) due to a prior therapy, unless returned to baseline or Grade 1 or less;
3. Major surgical intervention or participation in a therapeutic clinical trial within 28 days from Day 1 of the first dose of TTI-101;
4. Significantly impaired cardiac function such as: unstable angina pectoris, congestive heart failure with New York Heart Association (NYHA) class III or IV, myocardial infarction within the last 12 months prior to trial entry, signs of pericardial effusion, serious arrhythmia (including QTc prolongation of >470 ms and/or pacemaker) or prior diagnosis of congenital long QT syndrome or left ventricular ejection fraction <50% on screening echocardiogram;
5. History of cerebral vascular accident or stroke within the previous 2 years;
6. Uncontrolled hypertension (>160/100mm Hg);
7. History of Grade 3 or 4 allergic reactions attributed to compounds of similar chemical or biologic composition as TTI-101 (hydroxyl-naphthalene sulfonamides);
8. Known active metastases in the central nervous system (unless stable by brain imaging studies for at least 1 month without evidence of cerebral edema and no requirements for corticosteroids or anticonvulsants);
9. History of difficulty swallowing, malabsorption, or other chronic gastrointestinal disease or conditions that may hamper compliance and/or absorption of the investigational product;
10. Known Human Immunodeficiency Virus (HIV);
11. Subjects with chronic hepatitis B virus (HBV) infection, unless screening viral load <100 IU/mL on stable doses of antiviral therapy. Note: Subjects with chronic hepatitis C virus (HCV) infection are allowed to enroll in the study, but do not have a defined maximum viral load requirement for study entry;
12. Legal incapacity or limited legal capacity;
13. Pregnant or lactating women;
14. Any other condition, which in the opinion of the investigator, might impair the subject’s tolerance of trial treatment, the safety of the individual subject, or the outcome of the trial;
15. Previous treatment of the current malignancy with a STAT inhibitor.
