# Supplementary material for: Phase I Trial of TTI-101, a First-in-Class Oral Inhibitor of STAT3, in Patients with Advanced Solid Tumors
Source: Clin Cancer Res. 2025 Jan 10;31(6):965–74. doi: 10.1158/1078-0432.CCR-24-2920 (PMC11911802; doi:10.1158/1078-0432.CCR-24-2920)
Supplement: Supplementary Table S1 — Representativeness of Study Participants. [file ccr-24-2920_supplementary_table_s1_suppst1.docx]

| **Supplementary Table S1.** Representativeness of Study Participants | | |
| --- | --- | --- |
| Cancer type | Advanced metastatic solid tumors | |
| Considerations related to: | | |
| Sex | | In 2024^1^, it is estimated that 1,029,080 new cases of cancer will occur in male patients and 972,060 new cases in female patients in the United States. Among these new cases, over 90% are expected to be solid tumors for both males and females^1^.​ |
| Age | | The median age of cancer diagnosis in the United States is 67 years. Cancer is most frequently diagnosed in individuals aged 65 to 74 years. The median age at death from cancer is 73 years^1^. |
| Race/ethnicity | | In the United States, cancer incidence rates are highest among American Indian/Alaska Native (AIAN) populations, followed by White and Black populations, and lowest among Asian American/Pacific Islander (AAPI) populations^1^. |
| Geography | | Cancer is the second leading cause of death across all age groups in the United States. However, for both male and female patients aged 60 to 79 years, cancer is the leading cause of death^1^. |
| Overall representativeness of this study | | The median (range) age in this study was 63 (33-78) years, similar to the age distribution in other advanced solid tumor studies and slightly lower than the median age of patients diagnosed with cancer in the US overall.  This study includes a balanced representation of male and female patients: 51.6% male and 48.4% female.  White patients compromised ~78% of the patients in this study, followed by Asian (~8%), and African American (~3%) patients. In addition, ~11% were of ‘other’ race, reported greater than one self-reported race or did not report race information.  Regarding ethnicity, the majority of patients were non-Hispanic/Latino (~72%), while ~25% of patients were Latino/Hispanic, and ~2% did not report their ethnicity.  This study was conducted at 2 sites in the US, potentially limiting the representativeness of racial and ethnic distributions compared with populations in other geographic areas. As a pan-tumor phase 1 study, a broad range of solid tumors was included. |

1. Siegel, R.L., Giaquinto, A.N. & Jemal, A. Cancer statistics, 2024. *CA Cancer J Clin* **74**, 12-49 (2024).
